# Stereotactic body radiotherapy in lung cancer: a contemporary review

**DOI:** 10.3389/pore.2024.1611709

**Published:** 2024-02-27

**Authors:** Emese Csiki, Mihály Simon, Judit Papp, Márton Barabás, Johanna Mikáczó, Kristóf Gál, David Sipos, Árpád Kovács

**Affiliations:** ^1^ Department of Oncoradiology, Faculty of Medicine, University of Debrecen, Debrecen, Hungary; ^2^ Doctoral School of Clinical Medicine, University of Debrecen, Debrecen, Hungary; ^3^ Faculty of Health Sciences, University of Pécs, Pecs, Hungary

**Keywords:** SBRT, NSCLC, lung cancer, review, radiotherapy

## Abstract

The treatment of early stage non-small cell lung cancer (NSCLC) has improved enormously in the last two decades. Although surgery is not the only choice, lobectomy is still the gold standard treatment type for operable patients. For inoperable patients stereotactic body radiotherapy (SBRT) should be offered, reaching very high local control and overall survival rates. With SBRT we can precisely irradiate small, well-defined lesions with high doses. To select the appropriate fractionation schedule it is important to determine the size, localization and extent of the lung tumor. The introduction of novel and further developed planning (contouring guidelines, diagnostic image application, planning systems) and delivery techniques (motion management, image guided radiotherapy) led to lower rates of side effects and more conformal target volume coverage. The purpose of this study is to summarize the current developments, randomised studies, guidelines about lung SBRT, with emphasis on the possibility of increasing local control and overall rates in “fit,” operable patients as well, so SBRT would be eligible in place of surgery.

## Introduction

Lung cancer is one of the most common tumor types worldwide and the leading cause of cancer-related deaths among both women and men [1]. There are two main types of lung tumors: NSCLC (non-small cell lung cancer) and SCLC (small cell lung cancer). The cases are approx. 84% NSCLC, while about 13% SCLC [1]. Histological subtypes of NSCLC are adenocarcinomas, squamous cell carcinomas, and large cell carcinomas. Given the different aggressiveness and speed of progression of the two main histological types, their treatment strategies are different [2]. The type of treatment is determined by the histology of the disease, its stage, and the patient’s status. Due to developments in recent years (screening and radiation therapy techniques), a slight decrease in mortality can be seen in NSCLC patients [3]. Ganti et al. made a cross-sectional epidemiological analysis and calculated the most recent data in terms of incidence, prevalence, and survival [4]. The incidence of all stages per 100,000 people decreased from 46.4 to 40.9 in the United States between 2010–2017. The advanced stage decreased slightly (21.7–19.6), while the incidence of stage I patients increased to 10.8–13.2. The overall prevalence rose to 198.3/100,000, possibly because more and more young patients are diagnosed these days [4] the 5-year survival data have improved compared to the previous ones; the most significant improvement is 14.7%–25.7% in stage I patients receiving only radiation treatment [4].

The stage determines the prognosis of the disease at the time of diagnosis. The stage is defined based on the AJCC 8th edition (American Joint Committee of Cancer) since 2016; the use of the different TNM systems must be considered in the results of previous studies [5] Stage I-II disease is localized only to the lung tissue; lymph node positivity appears in the ipsilateral hilus in stage IIB. Radiotherapy plays an essential role in the treatment of early and advanced stages of lung cancer for both curative and palliative purposes [6]. In the case of lymph node-negative NSCLC, traditional treatment includes surgical resection (preferably video-assisted surgery-VATS lobectomy), which has been the standard of care, providing superior overall survival compared to other techniques. At the time of diagnosis, most of the patients are either technically or medically counted as inoperable for different reasons, such as poor overall health condition, elderly age, lung function, and multiple comorbidities, such as COPD (chronic obstructive pulmonary disease), cardiac and metabolic dysfunctions. For such patients, the primary treatment modality was conventional fractionated 3D-based radiation therapy, albeit with lower effectiveness and higher toxicity than surgery [7]. Today, conventional radiation therapy used in the treatment of small NSCLC foci located at a safe distance from important mediastinal organs has been replaced by a more effective, higher fractionation dose (ultra-hypofractionation) treatment, stereotaxic radiation therapy (SBRT).

SBRT is a radiation therapy method that is now widely spread, during which a relatively small, well-defined malignant tumor is treated in a few fractions, with a high dose per fraction. It is a radiation therapy technique with image guidance that can deliver very high radiation doses (ablative doses) to the target (tumor) with steep dose gradients outside the target while sparing the nearby healthy tissues (called organs at risk—OAR) [8] During SBRT, the size of the safety margins around the tumor can be reduced, which was made possible by advanced image guidance. With this ablative radiation dose, we precisely kill cancer cells, but we must pay attention to the fact that the radiation biology of the treatment changes during extreme hypofractionation; the tumor cells and healthy tissues also behave differently than during conventional fractionation. During the standard fractionation, we take advantage of the different repair mechanisms of tumor and healthy cells [9] This non-invasive technique has shown excellent local control rates and overall survival, with lower toxicity rates compared to traditional radiation therapy [8]. Another advantage is that the treatment lasts for a short time (1-2 weeks) and is suitable for outpatient treatment (doesn’t need hospitalization) [9] Given the high fractional dose, careful and accurate delivery is the most important part of the treatment. Modern technological solutions must be used both in the planning and delivery.

Regarding the high age and comorbidity of lung tumor patients, as well as the raising number of stage I patients due to the development of screening tests, the introduction and use of effective treatment methods other than surgery are increasingly important. Surgery remains the gold standard of care for operable patients; however, SBRT is the treatment of choice for patients with early-stage medically inoperable NSCLC [10].

## SBRT vs. conventional radiotherapy for early-stage NSCLC

Before the implementation of SBRT, conventional radiotherapy was the mainstay treatment for medically inoperable early-stage NSCLC patients. With conventional fractionation, 60–70 Gy was delivered with a fractional dose of 1.8–2 Gy. However, conventional radiotherapy showed reasonable local control and survival rates, and it was associated with significant toxicities [11]. In the first randomized phase 2 trial (SPACE), a high-fraction dose 3 × 22 Gy SBRT regimen was compared with 70/2 Gy conventional radiotherapy. The results of a total of 102 patients after a median follow-up of 37 months showed no significant difference between the two groups either in terms of 3-year PFS (progression free survival) (SBRT: 42%, Conventional RT: 42%) or OS (overall survival) at 3 years (SBRT: 54%, Conventional RT: 59%). The authors separately note that they were surprised by the exceptionally good results of the conventional therapy. When analysing the study, it should be considered that 36%–37% of the patients (in either group) did not undergo histological verification, during the examination, only about 60% of the patients underwent PET-CT, and there was an imbalance between the two groups in terms of tumor size, furthermore the number of T2 tumors was twice as high in the SBRT group. In terms of side effects, there were significantly fewer and lower-grade side effects in the SBRT group; the most significant difference was in esophagitis and pneumonitis, but mild side effects were encountered in both groups. Given the time of patient selection (2007–2011), SBRT treatment was still in its infancy, and 4D CT was only used in a few patients [12] The results of the RTOG 09.02 CHISEL multicentre randomized prospective phase 3 study were published in 2019. Peripheral, medically inoperable Stage I patients were studied. Comparing standard-dose RT (66 Gy/2 Gy or 50 Gy/2.5 Gy) with patients receiving SBRT (3 × 18 Gy or 4 × 12 Gy), superior local control could be achieved with SBRT without developing serious side effects [13]. Based on a retrospective study of a large number of patients (497 patients, 525 lesions), the 3-year local failure rate was 34.1% with standard radiotherapy and 13.6% with SBRT. PS matching showed a significant improvement in OS for SBRT (38.9% vs. 53.1%) [14]. In the Ontario Clinical Oncology Group’s ongoing phase 3 randomized study (LUSTRE trial), the results of medically inoperable patients receiving SBRT (4 × 12 Gy or 8 × 7.5 Gy depending on localization) are compared with modest hypofractionated radiotherapy (60/4 Gy).

### Medically inoperable early NSCLC patients

The standard care for early-stage non-small cell lung cancer patients (NSCLC) was lobectomy, as this provided the best chances of cure. However, surgery is not suitable for many NSCLC patients in the early stage for various reasons, such as old age, general condition, impaired lung function, or multiple comorbidities. As such, these patients are generally categorized as “medically inoperable” [7]. Based on the patient’s suitability for surgery, the American College of Chest Physicians practice guidelines categorized lung tumor patients into standard risk, high risk, or inoperable categories [15]. 25% of lung tumor patients diagnosed at an early stage are medically inoperable [16]. The exact definition is variable by studies. For patients who are not suitable for lobectomy, sublobar resection is often recommended. However, in the case of these patients, the high risk of complications and the uncertain oncological outcome must be considered. 3 prospective trials were compared in 2013: RTOG 0236 with patients receiving SBRT, patients undergoing sublobar resection (ACOSOG Z4032), and a trial examining radiofrequency treatment (ACOSOG Z40033). The overall 90-day mortality was 0% for RTOG 0236, 2.4% for surgery, and 2% for radiofrequency ablation [17]. In a prospective phase 2 study, after 7 years of follow-up, the results of SBRT in medically inoperable patients were published in 2018. 65 patients received 4 × 12.5 Gy; PET CT was performed in all cases as part of the examination. 5- and 7-year PFS were 49% and 38.2%, and OS was 55% and 47%, respectively. In terms of local recurrence, there is an increase at 7 years. Therefore, it is important to follow up on the occurrence of local recurrence even after 5 years. In addition, second primary lung cancer (SPLC) developed in 18.5% of cases; due to the high incidence, it is important to confirm the newly appeared lesions in the lungs with histology. Grade 3 side effects occurred in 4.6%, and Grade 4-5 were absent. The average age of the patients was 72.1 years [18] The Japan Clinical Oncology Group’s prospective study (JCOG0403) included patients with operable and inoperable histologically confirmed NSCLC tumors smaller than 3 cm who received 4 × 12 Gy SBRT treatment. The definition of medically operable is if the expected FEV1 > 800 mL (forced exspiratory volume), PaO2 > 65 torr, and did not have severe cardiac disease or severe diabetes mellitus; if either is impaired, it is considered inoperable. In the case of 100 inoperable patients, the 3-year OS was 59.9%; in the case of operable patients, the 3-year OS was 76.5%. When evaluating the results, the high average age of the patients (median age 78-79) must be considered [19] The 5-year results of the RTOG 0236 study were published in 2018, 55 medically inoperable patients were selected and received 3 × 18 Gy SBRT treatment. The long-term results of the multicenter phase two study show that the 5-year disease-free survival is 25.5%, and cancer recurrence occurs most often in the untreated lobe. During follow-up (median follow-up of 48 months), locoregional and/or distant metastasis developed in 38% of patients. The development of dissemination depends on the T stage. In the case of T1, the 5-year disseminated recurrence is 18.2%, and in the case of T2, it is 45.5% [20].

### Medically operable NSCLC- lobectomy vs. SBRT

The impact of using SBRT in the treatment of early-stage NSCLC is notable. In addition to irradiating inoperable patients, SBRT has recently been proposed as an alternative treatment to surgery, even for medically and technically operable NSCLC patients who refuse surgery. Recent clinical studies showed that with the use of SBRT, similar survival rates can be achieved to surgery without invasiveness and fewer treatment-related complications [8, 21]. Early-stage NSCLC lung cancer is a curable disease, and for medically fit patients, surgery (lobectomy with lymphadenectomy) is the gold standard treatment [22]. In the past few years, in addition to the development of radiation therapy, the surgical technique has also been modernized, open surgery was replaced by VATS in patients with early lung tumors, after which the number of hospitalization days and complications decreased, and the oncological results remained similar [23, 24]. In the case of SBRT, lymph node sampling is not performed; in several studies, this “deficiency” is considered the cause of lower locoregional control [18]. Based on the national lung cancer audit, only 60.6% of early-stage patients in the UK have undergone surgery. It can be seen that a significant portion of patients are at higher risk of surgical complications [25] Several randomized trials have attempted to compare surgery with SBRT but failed to accrue (RTOG 1021, SABRTooth) [26]. Two randomized phase 3 studies (STARS, ROSEL) aimed to compare the results of surgery and SBRT in operable patients. However, these were closed early due to slow accrual. Chang et al. analysed the two trials and processed the data of a total of 58 patients. Both in terms of 3-year estimated OS (SBRT: 95%, lobectomy: 79%) and recurrence-free survival (SBRT: 86%, lobectomy: 80%), patients who received SBRT had better results. However, we must consider the small number of patients, the short follow-up time, and the lack of modern surgical technologies (e.g., video-assisted thoracoscopic surgery) [8]. In 2021, the long-term results of the STARS study were published, as the SBRT arm was re-accrued with a larger number of participants (80 patients). The results of the SBRT-receiving patients were compared with the cohort of patients who underwent VATS lobectomy and lymphadenectomy (80 patients). All patients underwent PET-CT during the examination. There were no Grade 4-5 side effects with SBRT; Grade 3 side effects occurred in 1 patient. SBRT 3-year OS was 91%, 5-year OS was 87%, in the case of VATS lobectomy, 3-year OS was 91%, and 5-year OS was 84%. Overall, in terms of OS, SBRT is non-inferior to surgery in operable patients. There was no significant difference between the two patient groups regarding 3- and 5-year PFS either (SBRT 80% and 77%, surgery 88% and 80% respectively). After lymphadenectomy, the incidence of occult pathological lymph nodes was 10%; these patients received adjuvant chemotherapy [26]. It is still necessary to carry out comparative studies using modern techniques, and long-term results are needed. However, due to the fundamentally different modalities, it is not possible to blind either patients or clinicians at treatment allocation [22]. A propensity-matched analysis was also performed, the results of which were published in 2012. Retrospectively, the data of 64 VATS cases and 64 patients receiving SABR were compared, and locoregional failure (LRF) was investigated. Recurrence was considered if it was within the operating bed/prior PTV or ipsilateral hilo-mediastinal lymph node metastasis appeared. The 3-year LRF was 93% in the SBRT group and 82% in the surgical group. There was no difference in OS. Notably, in nearly 50% of the patients (in both groups), no histological sampling of the lung foci was performed [21]. In a single-arm phase two study (RTOG 0618), 3 × 18 Gy were administered to stage I peripheral foci in NSCLC patients in good condition (medially operable). The median follow-up time was 48.1 months. The 4-year local control was 96%, and the 4-year OS was 56%. Grade 3 side effects occurred in 14%, and Grade 4 side effects did not occur [27]. In the US VALOR trial, which is a randomized phase 3 ongoing study, veterans are enrolled with operable early-stage NSCLC from 2017, and by 2020, the number of enrolled patients exceeded the total number of phase 3 trial patients so far. The results of this trial may help us in the future [28]. Another prospective trial that is still in progress and is scheduled to be completed in 2026 is the POSTILV phase 2 study, where radical resection is compared with SBRT for stage I patients, and one of the aims of the study is to assess whether SBRT, with the correct dose and technique, more effective than surgery [16]. The STABLE-MATES phase 3 trial will be completed in 2024, comparing sublobar resection with SBRT in high-risk operable patients [16].

### Importance of localization

For an SBRT treatment, establishing an indication and choosing the correct fractionation scheme, the localization of the lesions within the thoracic cavity is the most important. In terms of localization, we distinguish between peripheral, central, and “extremely central,” i.e., ultracentral lesions [29]. The difference between the localizations is determined by the distance from the centrally located critical organs (trachea, heart, main bronchi, great vessels, esophagus) [30].

#### Peripheral lesions

Lung lesions located at a safe (>2 cm) distance from the central OARs. The previously described studies were conducted with medically inoperable and operable peripheral lung foci. A peripheral lesion can be close to (<1 cm) or touch the chest wall, which requires special attention. Late side effects affecting the chest wall can be, for example, rib fracture and chest wall pain. Rib fractures usually develop more than 6 months after radiation therapy. Previous studies have shown that chest wall side effects are more likely to occur with higher fractional doses (10 Gy vs. 20 Gy). In a retrospective study, the data of 134 patients were examined; 7.5% of them developed Grade 1 or Grade 2 chest wall side effects, and a significant correlation was found for V30 and V60. If V30 reached 80 cm^3^, side effects developed in 55%; in case it reached 100 cm^3^, the ratio was 74%; if V60 was 15 cm^3^, side effects occurred in 69% of patients, and if V60 reached 20 cm^3^, the percentage was 88%. The size of the GTV (Gross Tumor Volume) and the distance of the tumor from the chest wall showed no correlation with chest wall side effects [31].

#### Central lesions

Tumors in which PTV (Planning Target Volume) overlaps with a virtually drawn isotropic 2 cm extension around the vital mediastinal organs (proximal bronchial tree, heart, esophagus, large vessels) [29]. Considering the proximity of essential OARs, SBRT treatment of centrally localized lung lesions requires more attention. Various Grade 3 side effects are likely to occur, e.g., bronchial stenosis, bronchial hemorrhage, carditis, esophagitis, etc., [32]. In the phase II study published in 2006, Timmerman and his team reported “excessive,” high-grade toxicity with SBRT treatment of central tumors in medically inoperable patients. T1 patients received 3 × 22 Gy, while T2 patients received 3 × 20 Gy. With a high two-year local control rate (95%), a high percentage of Grade 3-4 side effects (11%) occurred, especially in the case of hilar/pericentral tumors. SBRT-related death occurred in 6 patients after 0.6–19.5 months after treatment [33]. The 4-year results of the study were published by Fakiris in 2009; the median survival was 32.4 months, where the distribution of lesions by localization was re-evaluated according to RTOG 0236. High-grade toxicity, Grade 3–5, occurred in 10.4% of peripheral tumors and 27.3% of central tumors. A total of 5 of the 70 patients participating in the study had Grade 5 toxicity [34]. The results of a 5-fraction SBRT treatment were reported in 2019 from the United States. In this phase I/II study, the goal was to establish the maximum tolerated fractional dose (MTD) in the case of central tumors. The MTD for 5 fractions was 12 Gy, with high local control (89.4%) [35]. A European study examined the treatment of central tumors in 8 fractions with a fractional dose of 7.5 Gy; after 35 months of follow-up, Grade 3 side effects occurred in 4 cases out of 63 patients, and there were no Grade 4-5 side effects. The 3-year local control was 92.6% [36]. In 2018, Roach et al.’s prospective phase I/II study was published, in which 5 × 11 Gy was found to be safe for central tumors, and excellent local control could be achieved [32]. LungTech, a prospective multicenter phase II EORTC trial, is underway with an 8 × 7.5 Gy fractionation scheme with high-quality technical solutions. It will investigate the role of FDG PET-CT in monitoring tumor progression and assessing side effects [30]. Based on the evidence presented so far, the maximum 50–60 Gy seems to be optimal for the fractionation scheme of centrally located lung tumors, delivered in 5 fractions, but considering the nearby OARs, 8 × 7.5 Gy is also a suitable option [16].

#### Ultracentral lesions

By definition, lesions where the PTV overlaps with one of the critical central organs belong here. In the case of SBRT, the risk of developing Grade 4-5 side effects is high (e.g., fistula, hemoptysis, bronchopulmonary hemorrhage, etc.). Several retrospective institutional studies demonstrated the plausibility of SBRT treatment of ultracentral tumors with an acceptable toxicity rate. When analyzing the results, we must consider that in many cases, only tumors adjacent to the peribronchial tree were examined, and lesions near the esophagus, heart, or large intestine were excluded [37]. Chang et al. used a more fractionated scheme in case the OAR constraints could not be met; 4 × 12.5 Gy or 10 × 7 Gy were delivered with an acceptable side effect rate [38]. The SUNSET study was started in 2018 and is currently still ongoing; this is a phase 1 multi-institutional study to find the maximum tolerated dose for ultracentral lesions up to 2 years after treatment. NSCLCs smaller than 6 cm were enrolled, and the aim was to limit the occurrence of Grade 3–5 adverse events to <30%. The first dose level is 8 × 7.5 Gy (15 × 4 Gy and 5 × 15 Gy are also examined) [39]. In 2021, the results of the HILUS trial, which is a phase two Nordic multicenter study, were published. 65 patients with ultracentral tumors were examined. A high rate of toxicity was encountered when 8 × 7 Gy were delivered; 22 out of 65 patients (34%) developed Grade 3–5 side effects, of which 10 were possible Grade 5 toxicity. The most common G5 side effect was bronchopulmonary hemorrhage, which developed 2–22 months after SBRT treatments. The authors concluded that in the case of lesions located <1 cm from the main bronchus and trachea, the use of the 8 × 7 Gy fractionation scheme is dangerous and prohibited [40, 41]. However, the difference between different centers regarding treatment setup and safety margins must be considered [41]. Based on Chen’s 2019 systematic review (a total of 250 patients’ data), after SBRT treatment of ultracentral tumors, the probability of developing Grade 3–5 side effects is 10% on average, and the median treatment-related mortality is 5% [42]. The ISRS (International Stereotactic Radiosurgery Society) published a practical guideline in 2023, summarizing the studies published so far on this topic (27 studies, all but one retrospective). The most frequently used fractionation schemes are 5 × 10 Gy, 8 × 7.5 Gy, and 12 × 5 Gy; 96% of the studies used motion management, most often 4D-CT-ITV (Internal Target Volume). The lesions were considered ultracentral if the PTV overlapped with the proximal bronchial tree (PBT). High local control (LC) (1 year LC: 92%, 2 year LC: 89%), with low life-threatening toxicities (G5: 4%) were found. PBT maximum dose (Dmax) needs to be considered according to the data of the meta-analysis; less fatal toxicity is expected if the BED_3_ (Biologically effective dose) value of PBT Dmax is < 180 Gy. The BED value of the treatments was a significant predictor for the one-year local control, and a negative trend appeared depending on the tumor size (smaller size, higher local control). In the ISRS guideline, 8 × 7.5 or 15 × 4 Gy are recommended for ultracentral tumors, and the PBT Dmax BED_3_ can be <133–150 Gy. If there is endobronchial involvement, the use of the ablative dose is not recommended [43]. Using a modern MR-guided technique with daily plan adaptation can reduce the probability of high toxicities when administering a large fractional dose. In the ongoing ARO-2021-3 MAGELLAN phase I trial, the aim was to find the maximum tolerated dose (MTD) for MR-guided SBRT in ultracentral localization for primary and secondary lung tumors from the 10 × 5.5 Gy scheme (_BED10_ = 85 .25 Gy) up to the 10 × 6.5 Gy scheme (BED_10_ = 107.25 Gy) [44].

### Dose schemes—BED

In 2015, Guckenberger et al. summarized the most common fractionation schemes of SBRT treatments used in lung tumors. Stereotactic body radiotherapy treatments were performed with a dose of 5–34 Gy per fraction in 1–10 fractions; comparing the biologically effective dose of the treatment regimens at alpha/beta 10, more than 200%–300% differences were found [45]. Several studies proved that increased biological effective dose increases local control. During SBRT planning, we must consider the expected toxicity and the oncological outcome to choose the appropriate fractionation. Based on the literature data, it is established that if BED< 100 Gy, low local control is expected [46]. Compared to conventional radiotherapy, SBRT with BED >100 Gy reduces local failure and may also increase overall survival [45, 47]. In 2007, during the Japanese retrospective multi-institutional study, 257 patients’ data and the details of the hypofractionated radiation treatments were examined, based on which a difference in 5-year overall survival was observed (BED_10_ < 100 Gy: 30.2%, BED_10_ > 100 Gy: 70.8%) [48]. The M.D. Anderson Cancer Center 2019 published the results of a retrospective study, where the data were obtained from the national cancer database, and the SBRT of maximum T2a tumors was compared. Radiation treatments were divided into two groups according to BED_10_: LowBED: 100–129 Gy and High BED: >130 Gy. Based on the aggregated results, the 5-year OS was 26% in the Low BED group and 34% in the High BED group. The study’s results suggest that a higher OS can be achieved with a higher BED above 130 Gy. It is important to emphasize that the exact localization of the lesions was not determined, which is an important factor for OS [49]. Examining small T1 and T2 tumors separately, Koshy et al. found no OS benefit for small tumors >150 Gy BED, which may suggest that we can achieve high OS in small tumors with BED_10_ 100–150 Gy [50]. When choosing the fractionation scheme, the localization of the lesion and its proximity to the various critical organs must be taken into account. In the case of small peripheral lesions, extreme hypofractionation schemes have proven to be effective and safe. In the RTOG 0915 prospective randomized phase 2 study, two regimens (1 × 34 Gy or 4 × 12 Gy) were compared for small peripheral lesions. Out of 84 patients, 10.3% received a single fraction, and 13.3% of the other group had at least G3 side effects within 1 year. The 2-year local control data was 97% and 92.7%, respectively. There was no difference between the two arms regarding two-year OS and DFS [15]. Based on the long-term results of this study (after a median follow-up of 4 years), no difference was found in late G3-G5 side effects. The 5-year progression-free survival in the single fraction group was 19.1%, and in the 4-fraction group, 33.3%, there was no significant difference. Therefore, it was established that there was no significant difference in the toxicity, the 5-year local control, or the 4-year survival data [51]. Another prospective trial compared single-fraction lung SBRT (1 × 30 Gy) with a 3-fraction (3 × 20 Gy) variant, also examining small peripheral lesions. The results of this phase 2 study were published in 2019. After a median follow-up of 53.8 months, out of 98 patients, Grade 3 or more severe side effects were described in 16% of the patients receiving one fraction and 12% in the group receiving 3 fractions. There were no differences in the survival and local control results [52]. Comparing the different fractionations, especially the less frequently used single fraction SBRT, using advanced planning techniques and image guidance is important to ensure safety. In the case of small, peripheral, early-stage NSCLC, SBRT given in 3–5 fractions is common. However, in 2022, a literature summary was published regarding SBRTs applied in one fraction, which may have the advantage of, e.g., fewer clinic visits [53]. This advantage could also be used during the COVID-19 pandemic, related to this, the ESTRO-ASTRO consensus was published in 2020. During the COVID-19 pandemic, 1 × 30–34 Gy was strongly considered if a small (<2 cm) lesion is located >2 cm from critical mediastinal organs and is also >1 cm from the chest wall [54]. If we use only one fraction, it must be considered that the BED used to determine the effectiveness of multi-fraction regimens cannot be applied due to radiobiological differences. Therefore, an SFED (single fraction equivalent dose) concept was created based on a new linear quadratic-linear model [55]. Besides choosing the exact fractionation scheme, it is important to take into account the dose prescription. Previous studies show that the PTV maximum dose correlates with the local control [56]. In his summary, Guckenberger points out the differences for peripheral tumors between the dose prescriptions defined in the studies: 60%–90% isodose line encompassing the PTV, and for SBRT treatments using the multi-fraction regimen, the PTV max dose was between 38–57 Gy [45]. BED values for the different SBRT schemes are listed in Table 1.

**TABLE 1:** BED_10_ values corresponding to different SBRT fractionations.

BED_10_ (Gy)	SBRT fractionation scheme (number of fractions × fractional dose [Gy])
100	5 × 10
106	4 × 12
113	4 × 12,5
132	5 × 12
151	3 × 18
180	3 × 20

### Tumor size (>5 cm)

By definition, SBRT means the treatment of small-sized tumors with a high fractional dose, but for now, the exact definition of the maximum size limit remains a question of debate. In the case of early, peripheral, medically inoperable lung tumors, early prospective studies proved to be successful treatment options where the size of the tumors was smaller than 4-5 cm. Retrospective studies have also found differences in overall survival according to tumor size. In the case of ultracentral tumors, the 5-year survival was determined by the size of the PTV, if the PTV <53 cm^3^ 61.6%, if PTV >53 cm^3^ 37.4%, respectively [57]. There is limited information from prospective randomized trials on large lung tumors treated with SBRT. In the multi-institutional retrospective study with the most significant number of patients (92 patients), 5–7.5 cm tumors were treated with 5 × 10 Gy with 2-year LC 73.2% and 2-year OS 46.4%. The OS correlated with the SUVmax (standardized uptake value) measured on the pre-treatment PET-CT. Distant failure occurred more often after treatment than local failure [58]. In the case of peripheral lung tumors larger than 5 cm, SBRT treatment can be administered with due caution, considering other treatment options and taking into account that lower local control and lower OS can be achieved compared to small lesions.

### Simulation

CT imaging needs to be prepared after staging, establishing the indication, and planning the session. The planned radiation therapy technique and motion management determine positioning during the simulation CT. Reproducibility is an important aspect when choosing the patient’s treatment position and positioning systems and ensuring small safety zones ideal for stereotactic treatment of the target volume. The total volume of the OARs in the thorax and the subsequent total planning target volume must be visible on the simulation CT [59].

### Motion management

The introduction of stereotactic treatments (SRT) appeared in the treatment of intracranial tumors; the SRT of extracranial lesions was delayed because new problems had to be dealt with, such as physiologic motion and the precise definition of the target volume [9]. Intrafractional movement that occurs during treatment, such as deviations resulting from breathing, can result in anatomical changes of several centimeters [47]. When performing SBRT of the lung, the “movement” of the tumor must be taken into account so that the planned dose is delivered to the right place and the surrounding tissues can be adequately protected. An essential and unmissable element of this is IGRT (image guided radiotherapy) before or during each faction. When selecting the IGRT method, we must consider the technique we will use and the treatment machine (linear accelerator, cyberknife). MV or kV imaging (3D CBCT, 4D CBCT, orthogonal x-ray) can be used as IGRT or optical verification, e.g., surface guided radiotherapy (SGRT). According to the phases of the respiratory cycle, the lesion in the lung changes its position. Motion management can be done in free breathing with motion-encompassing methods [60]. The most used is 4D CT, where we determine the breathing cycle and divide it into phases according to either the time phase or the amplitude. Several techniques can be used with 4D planning CT, such as internal target volume (ITV) determination or mid-ventilation determination. ITV is created from the union of GTVs delineated on CT slices of at least 3 breathing phases (max exhale, max inhale, and intermediate phase). Maximum intensity projection (MIP) can also be used to determine ITV. Comparing the two ITV approaches, it was found that ITV created with MIP is smaller compared to the GTV-based ones; in this case, tumor-miss may occur, although the MIP requires a shorter time [54]. Under free-breathing, real-time tracking is also a method of cyberknife [61]. Treatment in a specific part of the breathing cycle reduces the size of the target volume. Gating has been employed in some institutions to control respiratory motion [62]. Such gated methods can be ABC (active breath control) when the patient’s exhalation is inhibited after determining the breathing volume with the help of a spirometer. Another method is the deep inspiration breath hold (DIBH), when the patient holds the air in a certain deep inhalation level for about 10–15 s, most often in a voluntary manner (with audio-visual feedback), during which the pre-treatment verification takes place (it can be kV imaging or SGRT) and also delivery. Forced shallow breathing can be done with abdominal compression. In the case of tumor tracking, with the help of external or internal markers, the treatment is delivered only in certain breathing phases using intrafractional IGRT [60, 61]. The proper implementation of image-guided positioning and motion-management techniques can significantly reduce planning margins necessary for planning target volumes and, hence, the dose to the surrounding normal lung tissues [62].

### Target volume determination

Before radiation treatment of lung tumors, we define the target volumes using the ICRU 62 (Interational Commission on Radiation Units and measerments) and ICRU 83 definitions. Using contrast-enhanced diagnostic chest CT and PET-CT fusion, the GTV is first determined on the planning CT based on the visible extent of the tumor. The extension of GTV depends on the type of tumor according to the microscopic spread and the characteristics of the tumor (spread to surrounding organs), thus creating the CTV (clinical target volume). For SBRT treatments, 4D simulation is often performed, considering the changing tumor movement during the breathing cycle. In this case, the breathing cycle is divided into several phases, and GTVs must be defined separately in the phases. An ITV is obtained by summing the GTVs from all respiratory motion phases instead of CTVs, and the PTV is then obtained by applying a margin to the ITV to account for the setup and positioning uncertainties [6, 62].

### Dose constraints

When assessing radiation therapy plans, in addition to the coverage of the target volume and the use of adequate doses, we must pay close attention to the doses to the surrounding critical organs to reduce the development of early and late side effects. This way, we can ensure that SBRT is safe. In contrast to conventional radiotherapy, during SBRT, a high fractional dose is delivered in a few fractions, so the fractional dose received by the OARs is higher as well; therefore, we cannot use the restrictions defined for conventional radiotherapy [63]. Based on trials and published reports related to SBRT, the relevant dose constraints are determined, and due to the increasing number of treatments, these are frequently updated. When treating lung lesions, the OARs of the thorax must be considered (lung, heart, pericardium, esophagus, trachea, main bronchi, chest wall, brachial plexus, and liver, depending on localization). Most of the dose constraints for SBRT treatment are defined for 3, 5, and 8 fractions, but due to the use of the single fraction, which is becoming increasingly popular, the latest guidelines also help us with 1 fraction [64]. Depending on whether an organ is parallel (e.g., lungs) or serial (e.g., spinal cord), different parameters must be used in the treatment planning [59]. According to the latest data, the maximum dose is relevant to 0.1 cc for serial organs. In the case of parallel organs, it is crucial to determine, in addition to the maximum critical volume, the minimal volume that receives above the threshold dose (“minimum critical volume-cold constraints”) [64]. One of the first extensive summaries that defined the dose constraints used in SBRT is the AAPM task group was 101st in 2010. Here, the max dose applied to <0.35 cc, and there were no restrictions on the SBRT delivered in 8 fractions [59]. The constraints defined in the RTOG0813 trial can be used for 5 fraction lung SBRTs [35]. Based on a review published in 2021, the purpose is to systemically pool several published peer-reviewed clinical datasets and extract them in a clinically valid format [65]. The UK consensus guideline issued new guidelines 2022 based on updated data [64].

### Treatment delivery

Given that a high dose is delivered during SBRT, maximizing the protection of the organs at risk, in addition to the exact target volume coverage, with highly conformal dose distribution (even in the case of complicated, inhomogeneous anatomy) is crucial. This is one of the reasons for the advancement of modern delivery techniques in SBRT planning. Such techniques include intensity-modulated radiotherapy (IMRT) and volumetric-modulated arc therapy (VMAT) [66, 67]. Lung SBRT VMAT plans of 218 patients were compared to the previously more common 3D conformal technique. In terms of dose conformity/target coverage, there was no difference (V95% > 95% with both techniques); however, from the point of OARs, the dose constraints of the ipsilateral lung (V5, V10, V20, and MLD) were much easier to comply with in the case of VMAT technique. By using the FFF (flattening filter free) mode, the radiation treatment time can be significantly reduced; the delivery of 12 Gy in the case of FFF is approximately 1.5 min, and with FF, it is 8.3 min. The length of the radiation treatment is a very important aspect of radiation treatments using a high dose per fraction, as the patient must lie motionless throughout due to precise targeting [67].

### The importance of a PET-CT during examination and planning

In addition to biopsy, PET-CT also provides metabolic data and is essential in examining NSCLC patients. It helps establish a diagnosis and is also used in planning radiation therapy, including high-dose SBRT treatment [68–70]. The PET-CT performed for planning often verifies a stage change compared to the previous imaging, which is also vital before lung SBRT treatments to exclude unknown novum distant metastases and locoregional pathological lymph nodes. In 2014, 47 NSCLC patients were examined, and the results of PET-CT performed as part of staging were compared with those of planning PET-CT. A new locoregional or distant metastasis was found in 51% of the patients. In the study, it was determined that if 6 weeks pass between the two PETs, the treatment of the disease changes in 26% of patients due to upstaging [71]. In finding pathological mediastinal lymph nodes, PET-CT has higher accuracy rates than diagnostic chest CT. The fact that different radiation therapists can delineate a target volume of much more similar size and shape to each other due to the metabolic data is of great help in radiation therapy planning, especially in determining the GTV. A good correlation was found when comparing the tumor sizes determined based on PET-CT with the pathological sizes after the subsequent surgery [68, 70] The extent of FDG (fluorodeoxyglucose) accumulation within the tumor is most often determined by the SUVmax (standardized uptake value). Several studies have proved the predictive value of the SUVmax before treatments on local control; with an SUVmax of >3, local or distant failure is more likely to occur [72]. Chang examined 130 patients after 4 × 12.5 Gy was given to peripheral small lesions (<T1) and found that if the SUVmax on the staging PET-CT was below 6.2, a significantly higher OS was expected. The staging PET-CT was the only independent significant predictor for OS [73]. After SBRT treatment, PET-CT can also help with the question of recurrence-fibrosis arising on chest CT. If the SUVmax is > 5 on the 12-week PET-CT after treatments, it is more likely to indicate recurrence [72] In ongoing trials, they also investigate the effect on local control if the dose is escalated to areas with a high SUVmax within the tumor [68].

### Guidelines

The European guidelines for treating early-stage NSCLC patients have not been updated since 2014–2017. The results of the currently ongoing, critical phase 2 and 3 studies are still in progress, which can determine the place of SBRT compared to lobectomy and will provide additional help in choosing the appropriate fractionation.

1. ESMO (European Society of Medical Oncology—2014): From the point of view of the feasibility of the surgery (to estimate the morbidity and mortality that may occur after the operation), the patients must be grouped in terms of risk; a cardiac and pulmonary functional assessment is required for this evaluation. The recalibrated thoracic revised cardiac risk index (RCRI) determines cardiac high risk. The case is of low risk regarding respiratory function if both FEV1 and DLCO are >80%. In the case of invasive NSCLC, the gold standard treatment is still lobectomy; lymph node dissection is not mandatory in all cases (it can be omitted if it is cN0 on PET-CT). If lobectomy cannot be performed, SBRT treatment should be chosen. In terms of fractionation, delivery, and motion management it is not covered by the ESMO guidelines. Even in multifocal cases, surgery is the primary choice; SBRT is only chosen if surgery cannot be performed [74].
2. ASTRO (American Society for Radiation Oncology—2017): SBRT can be offered as an alternative to lobectomy for “high risk” peripheral early-stage NSCLC patients, but in the case of “standard risk,” operable patients, SBRT can only be recommended in a clinical trial as an alternative to surgery, considering that long-term side effect and survival data >3 years are still missing. SBRT is recommended in medically inoperable cases. In central localization, at least 4 fractions are recommended, but in the case of a higher risk of side effects, 6–15 fractions are also possible. SBRT can also be given in histologically unverified cases. After pneumonectomy, SBRT is recommended for novum lung cancer [75].
3. NCCN (National Comprehensive Cancer Network—2023.3): The use of advanced technologies is important for precise, high-dose curative RT (planning 4D CT, PET-CT fusion, IMRT/VMAT screening, motion management, appropriate high-level IGRT). For medically operable patients, lobectomy plus mediastinal lymph node dissection is recommended. For high-risk patients (from a surgical point of view), SBRT is a suitable alternative to lobectomy. For medically inoperable early-stage NSCLC patients, SBRT is recommended for tumors smaller than 5 cm if the constraints of the surrounding organs permit. Based on tumor localization, the recommended fractionation: small peripheral: 1 × 25–34 Gy, peripheral tumors: 3 × 18–20 Gy, central and peripheral tumors smaller than 5 cm: 4 × 12 Gy, 4 × 12.5 Gy, 5 × 10–10.5 Gy, for central lesions 8 × 7.5 Gy. According to the NCCN guideline, SBRT can be performed with a higher number of fractions (max. 10 fractions) in the case of ultracentral location. Treatment in 3 or fewer fractions is prohibited for central and ultracentral tumors. Dose constraints for healthy organs were determined based on RTOG 0618, 0813, and 0915 [6].

### SBRT treatment and immunotherapy

During SBRT treatment, a high dose is delivered per fraction, which helps to achieve high local control in patients with early-stage lung tumors. The probability of the appearance of distant metastases is 10%–20%. The increased effect of the immune system may cause out-of-field tumor regression during radiation therapy [29]. The reason for this is that the release of tumor antigens increases due to the ionizing radiation, and thus, the adaptive immune system recognizes them more easily; this is called the abscopal effect [76]. During SBRT, we detect tumor shrinkage, but tumor cell fragments are also present and can behave immunogenically. It is of therapeutic importance that, in the case of lung tumors, an exceptional antitumor immune response is developed to target tumor antigens. The immune response can be inhibited by regulating different checkpoint pathways. For example, antibodies against PD1 and CTLA-4 can increase the antitumor effect [29]. Combined, SBRT and immunotherapy can be synergistic, effective, and safe treatments [77]. In advanced NSCLC patients, it was investigated that progression-free survival and overall survival were higher if the patients also received radiotherapy before immunotherapy [78]. Immunotherapy with SBRT treatment resulted in a good clinical response in melanoma and advanced lung tumor patients [79, 80]. PD-1/PD-L1 inhibitors such as pembrolizumab and atezolizumab are recommended as first-line treatment in advanced NSCLC patients with high PD-L1 expression [77]. A higher immunological antitumor effect can be achieved with a higher fractional dose than conventional radiotherapy. The SBRT treatment creates a supportive immune microenvironment for subsequent immune checkpoint inhibitors; in return, the immune checkpoint inhibitors reduce radiation resistance and boost the abscopal effect [77]. Administering immunotherapy (immune checkpoint inhibitors—ICI), enhancing the immune response, together with SBRT treatment decreases the probability of developing regional and distant metastases, so, e.g., in the case of central-ultracentral lung tumors, safer, reduced-dose SBRT treatment should be considered (BED<100 Gy) [29]. Currently, several studies are ongoing that investigate the administration of SBRT and ICIs in early-stage NSCLC; in terms of dose, there is a lack of consensus, but the most frequently studied ICIs are pembrolizumab, atezolizumab, and durvalumab [77, 81].

### Radiological and pulmonary function changes after SBRT

CT and PET-CT are used for control purposes after SBRT treatments. Given that more and more “fit” patients are receiving this form of treatment, it is crucial to define the follow-up protocol precisely due to the more prolonged survival. Detecting local recurrence is often difficult because its radiological picture can be confused with early and late lung injury after SBRT. Side effects are considered early if they develop sooner than 6 months after radiation treatment (pneumonitis-consolidation, GGO) and late (fibrosis). In 2012, a systemic literature review summarized possible radiological changes after SBRT. Considering the conformal dose distribution at high doses, the resulting CT abnormalities are different from the sharp-bordered lesions corresponding to the fields seen after conventional radiation treatments. During SBRT, the lung volume receiving smaller doses will be larger [82]. The phase 3 CHISEL study compared conventional (mean BED_10_: 65.49 Gy) and SBRT (mean BED_10_: 125.92 Gy) treatment of medically inoperable patients with small lung cancer patients. The analysis of pulmonary function changes was published in 2022. Despite the significant BED difference, no significant difference was found after a 3- to 12-day follow-up between the patients’ PFT (pulmonary function test), nor in FVC, DLCO, and FEV1 [83]. Overall, acute side effects developed in 62% of cases; consolidation is visible in almost half of the cases. Among the radiological changes on thorax CT, the most common indication of recurrence is enlarging opacity after 1 year. The blurred border, the examined area’s inhomogeneity, and the air bronchogram’s disappearance can be suspicious [82]. Chang et al. examined the data of 130 patients who received 4 × 12.5 Gy for stage I peripheral NSCLC lung lesions. 9.3% of patients experienced Grade 2 pneumonitis and 2.3% Grade 3 pneumonitis. After multivariate analysis, it was determined that the probability of developing pneumonitis is significantly higher if the mean ipsilateral lung dose (MLD) > 9.14 Gy [73]. In 2014, new statistical and geometric analysis methods were used to examine the dosimetric parameters that can affect the development of lung injuries. The planned CT data of 24 patients were compared (with deformable registration) with the diagnostic thorax CT images taken at 3, 6, and 12 months (after SBRT treatment). The patients received 3 × 12–18 Gy or 4 × 12.5 Gy. There was no Grade 3-4 pneumonitis, Grade 2 in 15% of the patients. The critical dose (low-dose peak location) of lung radiographic injury was approximately 35 Gy (with a standard deviation of 10 Gy), or 70% of the prescribed dose. The larger the PTV, the smaller the critical dose. Therefore, in the case of a larger PTV, the probability of developing pneumonitis/fibrosis is higher [84]. Radiological changes do not always correlate with decreased pulmonary function. Pulmonary function deterioration occurs very rarely after SBRT treatment of peripheral lesions. Analysing the phase 2 RTOG 0236 study, the data of 55 medically inoperable patients who received 3 × 18 Gy treatment were examined. They found that poor respiratory function before treatment did not increase the likelihood of developing pulmonary toxicity, and patients who became inoperable for cardiac reasons had a lower 2-year OS. After SBRT treatment, Grade 0 and 1 PFT (pulmonary function test) changes were observed in most patients based on the RTOG SBRT pulmonary scale; > 70% did not change PFT [85].

### MRI-guided lung SBRT

The use of MR in stereotactic treatments is novel. Currently, the most used image-guided strategies are techniques utilizing X-rays. The introduction of MRI-guided techniques can facilitate the isolation of different soft tissues. Compared to X-ray imaging, radiation exposure is also an important aspect. MRI can help us in many ways when performing lung SBRT, for example, by defining target volumes, planning, and using motion management. Intra- and inter-fractional deviations can be easily recognized and eliminated [47]. A clear advantage can be seen during MRI-guided adaptive radiation treatment of central lesions (SMART). After performing a breath hold 3D MRI simulation and planning CT, the plan is prepared using an inverse technique (IMRT, VMAT). Before each fraction, a 3D MRI image of the position to be treated per the anatomy of the day is taken. After the rigid registration with the gross tumor volume on the planning MRI scan, a couch shift is performed, if necessary. The OAR contours are propagated to the chosen MRI scan of the day using deformable image co-registration. The clinician then makes the necessary corrections on the GTV, and the PTV is generated with an isotropic margin of 5 mm. The online plan is then reoptimized with the same optimization objectives and field parameters. Based on real-time 2D MR images, the degree of breath-hold (gating window) can be monitored during fraction. Due to the central localization, the proximity of the OARs complicates the implementation of SBRT. Using on-table plan adaptation reduced the OAR planning constraint violations (*p* < .05), but the OAR maximum doses mainly remained stable. With the MRI-guided technique, we can reduce the size of the PTV during breath-hold gated treatments with the help of continuous lesion visualization. By increasing the coverage, the target of 95% PTV coverage was achieved in 95% of the plans, compared to the “predicted” plans (71%). As well as the daily adaptation of plans, they reduce the likelihood of side effects by monitoring the anatomical and target volume changes that occur during each fraction [86]. Finazzi et al. investigated the advantages of MRI-guided adaptive radiation therapy (SMART) during SBRT treatment of 25 peripheral lung lesions. Compared to free-breathing plans using 4D CT-based ITV, the size of SMART-PTVs has decreased, and the PTV coverage has improved. BED_10_ > 100 Gy can be delivered to PTV 95% in a higher percentage of patients, but the dosimetric benefits were modest. In conclusion, it can be said that in the case of peripheral lesions, it is not always beneficial to use SMART; it should be chosen if there is a higher probability of the development of severe side effects (if the lesion moves >1 cm, re-irradiation, previous lung surgery, severe lung disease in the anamnesis) [87]. When using this technique, we must remember that it takes longer to perform compared to CT-based and non-adaptive treatments (for peripheral lesions, on average, 48 min door-to-door; for central lesions, 59 min) [86, 87]. An ongoing prospective study, with phase 1 data reported to date, focuses on ultracentral tumors. Five lesions were examined, and 5 × 10 Gy were delivered while keeping the strict OAR constraints. 70% of the 25 fractions were based on an adapted plan due to the OAR violation. No Grade 3 acute side effects were found 6 months after treatment. In the future, the application of SMART may expand the SBRTs indicated for ultracentral tumors [88]. It remains a question whether the benefits provided by MR also represent a survival advantage and whether the cost of MR LINAC is worth it [47].

## Discussion

Nowadays, SBRT is an increasingly common and frequently used form of radiotherapy treatment. SBRT has revolutionized the early-stage NSCLC and oligometastatic cancer treatment paradigm, providing a highly effective and non-invasive alternative to surgery. This curative, safe treatment method can also be used for patients considered inoperable due to some comorbidity. Elderly patients with poor respiratory function can also be treated as it does not worsen PFT after treatment [85]. The conventional radiation treatment, previously chosen for medically inoperable patients, has now been replaced by SBRT if the localization of the tumor allows it [13, 14]. High-fractionation SBRT can be given safely to peripheral lesions, but the results of modest hypofractionation treatments for local control are still ongoing (LUSTRE study). The localization determines the dose of SBRT; tumors close to the chest wall and localized centrally are treated with a lower fractional dose in more fractions [31, 35]. SBRT treatment of ultracentral lesions is not recommended based on guidelines but can be carried out under safe conditions, as long as the dose of the nearby OARs is adequate and the fractional dose is as low as possible [37, 43]. The appropriate dose is still being determined (SUNSET study) [39]. The size of the lung lesion affects the expected local control and the development of dissemination [20]. The guidelines recommend SBRT treatment for less than 5 cm lung lesions, but tumors >5 cm can also be treated with appropriate care [58]. Comparing lobectomy and SBRT in the case of operable patients, previous studies have produced similar results [26], but prospective phase 2-3 studies are still ongoing (VALOR, POSTILV, STABLE-MATES) [16].

During the follow-up after SBRT treatment, PET-CT can be an additional examination of the diagnostic CT, helping to distinguish between fibrosis and recurrence. It indicates a relapse if, in addition to increasing opacity, the SUV max value is higher than 5 [72]. Future directions involve refining patient selection (for example, not only inoperably patients benefit from it), optimizing treatment planning (VMAT planning, MR guided therapies), and integrating SBRT with novel systemic therapies like immunotherapy.

## References

[1] American Cancer Society. Facts & Figures 2019. Am Cancer Soc [Internet] (2019):1–76. Available at: https://www.cancer.org/content/dam/cancer-org/research/cancerfacts-and-statistics/annual-cancer-facts-andfigures/2019/cancer-facts-andfigures .

[2] Travis WD Brambilla E Nicholson AG Yatabe Y Austin JHM Beasley MB The 2015 world health organization classification of lung tumors: impact of genetic, clinical and radiologic advances since the 2004 classification. J Thorac Oncol (2015) 10:1243–60. 10.1097/JTO.0000000000000630 26291008

[3] Howlader N Forjaz G Mooradian MJ Meza R Kong CY Cronin KA The effect of advances in lung-cancer treatment on population mortality. N Engl J Med (2020) 383(7):640–9. 10.1056/NEJMoa1916623 32786189 PMC8577315

[4] Ganti M Alyssa BK Ion C Seal B Chou E. Update of incidence, prevalence, survival, and initial treatment in patients with non-small cell lung cancer in the US. JAMA Oncol (2021) 7(12):1824–32. 10.1001/jamaoncol.2021.4932 34673888 PMC8532041

[5] Goldstraw P Chansky K Crowley J Rami-Porta R Asamura H Eberhardt WEE The IASLC lung cancer staging project: proposals for revision of the TNM stage groupings in the forthcoming (eighth) edition of the TNM Classification for lung cancer. J Thorac Oncol (2016) 11(1):39–51. 10.1016/j.jtho.2015.09.009 26762738

[6] NCCN. NCCN Guidelines Version 3. 2023 Non-small cell lung cancer (2023).

[7] Howington JA Blum MG Chang AC Balekian AA Murthy SC. Treatment of stage I and II non-small cell lung cancer: diagnosis and management of lung cancer, 3rd ed: american College of Chest Physicians evidence-based clinical practice guidelines. Chest (2013) 143(5):e278S–e313S. 10.1378/chest.12-2359 23649443

[8] Chang JY Senan S Paul MA Mehran RJ Louie AV Balter P Stereotactic ablative radiotherapy versus lobectomy for operable stage I non-small-cell lung cancer: a pooled analysis of two randomised trials. Lancet Oncol (2015) 16(6):630–7. 10.1016/S1470-2045(15)70168-3 25981812 PMC4489408

[9] Timmerman RD Herman J Cho LC. Emergence of stereotactic body radiation therapy and its impact on current and future clinical practice. J Clin Oncol (2014) 32:2847–54. 10.1200/JCO.2014.55.4675 25113761 PMC4152712

[10] Prezzano KM Ma SJ Hermann GM Rivers CI Gomez-Suescun JA Singh AK. Stereotactic body radiation therapy for non-small cell lung cancer: a review. World J Clin Oncol (2019) 10(1):14–27. 10.5306/wjco.v10.i1.14 30627522 PMC6318482

[11] Rowell NP Williams CJ. Radical radiotherapy for stage I/II non-small cell lung cancer in patients not sufficiently fit for or declining surgery (medically inoperable). Cochrane Database Syst Rev (2001) 2:CD002935. 10.1002/14651858.CD002935 11279780

[12] Nyman J Hallqvist A Lund JÅ Brustugun OT Bergman B Bergström P SPACE – a randomized study of SBRT vs conventional fractionated radiotherapy in medically inoperable stage I NSCLC. Radiother Oncol (2016) 121(1):1–8. 10.1016/j.radonc.2016.08.015 27600155

[13] Ball D Mai GT Vinod S Babington S Ruben J Kron T Stereotactic ablative radiotherapy versus standard radiotherapy in stage 1 non-small-cell lung cancer (TROG 09.02 CHISEL): a phase 3, open-label, randomised controlled trial. Lancet Oncol (2019) 20(4):494–503. 10.1016/S1470-2045(18)30896-9 30770291

[14] von Reibnitz D Shaikh F Wu AJ Treharne GC Dick-Godfrey R Foster A Stereotactic body radiation therapy (SBRT) improves local control and overall survival compared to conventionally fractionated radiation for stage I non-small cell lung cancer (NSCLC). Acta Oncol (Madr) (2018) 57(11):1567–73. 10.1080/0284186X.2018.1481292 PMC6508090 29873277

[15] Videtic GMM Hu C Singh AK Chang JY Parker W Olivier KR A randomized phase 2 study comparing 2 stereotactic body radiation therapy schedules for medically inoperable patients with stage i peripheral non-small cell lung cancer: NRG Oncology RTOG 0915 (NCCTG N0927). Int J Radiat Oncol Biol Phys (2015) 93:757–64. 10.1016/j.ijrobp.2015.07.2260 26530743 PMC4744654

[16] Rodríguez De Dios N Navarro-Martin A Cigarral C Chicas-Sett R García R Garcia V GOECP/SEOR radiotheraphy guidelines for non-small-cell lung cancer. World J Clin Oncol (2022) 13(4):237–66. 10.5306/wjco.v13.i4.237 35582651 PMC9052073

[17] Crabtree T Puri V Timmerman R Fernando H Bradley J Decker PA Treatment of stage i lung cancer in high-risk and inoperable patients: comparison of prospective clinical trials using stereotactic body radiotherapy (RTOG 0236), sublobar resection (ACOSOG Z4032), and radiofrequency ablation (ACOSOG Z4033). J Thorac Cardiovasc Surg (2013) 145(3):692–9. 10.1016/j.jtcvs.2012.10.038 23174176

[18] Sun B Brooks ED Komaki RU Liao Z Jeter MD McAleer MF 7-year follow-up after stereotactic ablative radiotherapy for patients with stage I non–small cell lung cancer: results of a phase 2 clinical trial. Cancer (2017) 123(16):3031–9. 10.1002/cncr.30693 28346656 PMC5544582

[19] Nagata Y Hiraoka M Shibata T Onishi H Kokubo M Karasawa K Prospective trial of stereotactic body radiation therapy for both operable and inoperable T1N0M0 non-small cell lung cancer: japan Clinical Oncology Group Study JCOG0403. Int J Radiat Oncol Biol Phys (2015) 93(5):989–96. 10.1016/j.ijrobp.2015.07.2278 26581137

[20] Timmerman RD Hu C Michalski JM Bradley JC Galvin J Johnstone DW Long-term results of stereotactic body radiation therapy in medically inoperable stage I non-small cell lung cancer. JAMA Oncol (2018) 4(9):1287–8. 10.1001/jamaoncol.2018.1258 29852036 PMC6117101

[21] Verstegen NE Oosterhuis JWA Palma DA Rodrigues G Lagerwaard FJ van der Elst A Stage I-II non-small-cell lung cancer treated usingeither stereotactic ablative radiotherapy (SABR) orlobectomy by video-assisted thoracoscopic surgery(VATS): outcomes of a propensity score-matchedanalysis. Ann Oncol (2013) 24(6):1543–8. 10.1093/annonc/mdt026 23425947

[22] Franks KN McParland L Webster J Baldwin DR Sebag-Montefiore D Evison M SABRTooth: a randomised controlled feasibility study of stereotactic ablative radiotherapy (SABR) with surgery in patients with peripheral stage I nonsmall cell lung cancer considered to be at higher risk of complications from surgical resection. Eur Respir J (2020) 56(5):2000118. 10.1183/13993003.00118-2020 32616595

[23] Yamamoto K Ohsumi A Kojima F Imanishi N Matsuoka K Ueda M Long-term survival after video-assisted thoracic surgery lobectomy for primary lung cancer. Ann Thorac Surg (2010) 89(2):353–9. 10.1016/j.athoracsur.2009.10.034 20103297

[24] Ilonen IK Räsänen JV Knuuttila A Salo JA Sihvo EI. Anatomic thoracoscopic lung resection for non-small cell lung cancer in stage i is associated with less morbidity and shorter hospitalization than thoracotomy. Acta Oncol (Madr) (2011) 50(7):1126–32. 10.3109/0284186X.2011.555780 21314296

[25] Onkologie A Daten D Breyer-kohansal R Hartl S Burghuber OC Urban M National Lung Cancer Audit annual report (for the audit period 2018). Eur Respir J (2021) 14:2103201.

[26] Chang JY Mehran RJ Feng L Verma V Liao Z Welsh JW Stereotactic ablative radiotherapy for operable stage I non-small-cell lung cancer (revised STARS): long-term results of a single-arm, prospective trial with prespecified comparison to surgery. Lancet Oncol (2021) 22(10):1448–57. 10.1016/S1470-2045(21)00401-0 34529930 PMC8521627

[27] Timmerman RD Paulus R Pass HI Gore EM Edelman MJ Galvin J Stereotactic body radiation therapy for operable early-stage lung cancer: findings from the NRG oncology RTOG 0618 trial. JAMA Oncol (2018) 4(9):1263–6. 10.1001/jamaoncol.2018.1251 29852037 PMC6117102

[28] Moghanaki D. Strategic initiatives for veterans with lung cancer. Fed Pract (2020) 37:S76–S80. 10.12788/fp.0019 32908355 PMC7473723

[29] Chang JY Bezjak A Mornex F, IASLC Advanced Radiation Technology Committee. Stereotactic ablative radiotherapy for centrally located early stage non–small-cell lung cancer: what we have learned. J Thorac Oncol (2015) 10:577–85. 10.1097/JTO.0000000000000453 25514807

[30] Adebahr S Collette S Shash E Lambrecht M Le Pechoux C Faivre-Finn C LungTech, an EORTC Phase II trial of stereotactic body radiotherapy for centrally located lung tumours: a clinical perspective. Br J Radiol (2015) 88(1051):20150036. 10.1259/bjr.20150036 25873481 PMC4628529

[31] Stephans KL Djemil T Tendulkar RD Robinson CG Reddy CA Videtic GMM. Prediction of chest wall toxicity from lung stereotactic body radiotherapy (SBRT). Int J Radiat Oncol Biol Phys (2012) 82(2):974–80. 10.1016/j.ijrobp.2010.12.002 21300453

[32] Roach MC Robinson CG DeWees TA Ganachaud J Przybysz D Drzymala R Stereotactic body radiation therapy for central early-stage NSCLC: results of a prospective phase I/II trial. J Thorac Oncol (2018) 13(11):1727–32. 10.1016/j.jtho.2018.07.017 30056162

[33] Timmerman R McGarry R Yiannoutsos C Papiez L Tudor K DeLuca J Excessive toxicity when treating central tumors in a phase II study of stereotactic body radiation therapy for medically inoperable early-stage lung cancer. J Clin Oncol (2006) 24(30):4833–9. 10.1200/JCO.2006.07.5937 17050868

[34] Fakiris AJ McGarry RC Yiannoutsos CT Papiez L Williams M Henderson MA Stereotactic body radiation therapy for early-stage non-small-cell lung carcinoma: four-year results of a prospective phase II study. Int J Radiat Oncol Biol Phys (2009) 75(3):677–82. 10.1016/j.ijrobp.2008.11.042 19251380

[35] Bezjak A Paulus R Gaspar LE Timmerman RD Straube WL Ryan WF Safety and efficacy of a five-fraction stereotactic body radiotherapy schedule for centrally located non-small-cell lung cancer: NRG oncology/RTOG 0813 trial. J Clin Oncol (2019) 37:1316–25. 10.1200/JCO.18.00622 30943123 PMC6524984

[36] Haasbeek CJA Lagerwaard FJ Slotman BJ Senan S. Outcomes of stereotactic ablative radiotherapy for centrally located early-stage lung cancer. J Thorac Oncol (2011) 6. 10.1097/JTO.0b013e31822e71d8 21892102

[37] Chaudhuri AA Chen K Diehn M Loo BW. Stereotactic ablative radiotherapy for central and ultra-central lung tumors. Ther Radiol Oncol (2019) 3:18. 10.21037/tro.2019.05.01 33880444 PMC8054989

[38] Chang JY Li QQ Xu QY Allen PK Rebueno N Gomez DR Stereotactic ablative radiation therapy for centrally located early stage or isolated parenchymal recurrences of non-small cell lung cancer: how to fly in a “no fly zone.”. Int J Radiat Oncol Biol Phys (2014) 88(5):1120–8. 10.1016/j.ijrobp.2014.01.022 24661665

[39] Giuliani M Mathew AS Bahig H Bratman SV Filion E Glick D SUNSET: stereotactic radiation for ultracentral non–small-cell lung cancer—a safety and efficacy trial. Clin Lung Cancer (2018) 19(4):e529–32. 10.1016/j.cllc.2018.04.001 29759332

[40] Rosenberg SA Mak R Kotecha R Loo BW Senan S. The nordic-HILUS trial: ultracentral lung stereotactic ablative radiotherapy and a narrow therapeutic window. J Thorac Oncol (2021) 16:e79–80. 10.1016/j.jtho.2021.06.030 34561039

[41] Lindberg K Grozman V Karlsson K Lindberg S Lax I Wersäll P The HILUS-trial—a prospective nordic multicenter phase 2 study of ultracentral lung tumors treated with stereotactic body radiotherapy. J Thorac Oncol (2021) 16(7):1200–10. 10.1016/j.jtho.2021.03.019 33823286

[42] Chen H Laba JM Zayed S Boldt RG Palma DA Louie AV. Safety and effectiveness of stereotactic ablative radiotherapy for ultra-central lung lesions: a systematic review. J Thorac Oncol (2019) 14:1332–42. 10.1016/j.jtho.2019.04.018 31075543

[43] Yan M Louie AV Kotecha R Ashfaq Ahmed M Zhang Z Guckenberger M Stereotactic body radiotherapy for ultra-central lung tumors: a systematic review and meta-analysis and international stereotactic radiosurgery society practice guidelines. Lung Cancer (2023) 182:107281. 10.1016/j.lungcan.2023.107281 37393758

[44] Regnery S Ristau J Weykamp F Hoegen P Sprengel SD Paul KM Magnetic resonance guided adaptive stereotactic body radiotherapy for lung tumors in ultracentral location: the MAGELLAN trial (ARO 2021-3). Radiat Oncol (2022) 17(1):102. 10.1186/s13014-022-02070-x 35614486 PMC9134672

[45] Guckenberger M. Dose and fractionation in stereotactic body radiation therapy for stage i non-small cell lung cancer: lessons learned and where do we go next? Int J Radiat Oncol Biol Phys (2015) 93:765–8. 10.1016/j.ijrobp.2015.08.025 26530744

[46] Onishi H Araki T Shirato H Nagata Y Hiraoka M Gomi K Stereotactic hypofractionated high-dose irradiation for stage I nonsmall cell lung carcinoma: clinical outcomes in 245 subjects in a Japanese multiinstitutional study. Cancer (2004) 101(7):1623–31. 10.1002/cncr.20539 15378503

[47] Menten MJ Wetscherek A Fast MF. MRI-guided lung SBRT: present and future developments. Physica Med (2017) 44:139–49. 10.1016/j.ejmp.2017.02.003 28242140

[48] Onishi H Shirato H Nagata Y Hiraoka M Fujino M Gomi K Hypofractionated stereotactic radiotherapy (HypoFXSRT) for stage I non-small cell lung cancer: updated results of 257 patients in a Japanese multi-institutional study. J Thorac Oncol (2007) 2. 10.1097/JTO.0b013e318074de34 17603311

[49] Moreno AC Fellman B Hobbs BP Liao Z Gomez DR Chen A Biologically effective dose in stereotactic body radiotherapy and survival for patients with early-stage NSCLC. J Thorac Oncol (2020) 15(1):101–9. 10.1016/j.jtho.2019.08.2505 31479748

[50] Koshy M Malik R Weichselbaum RR Sher DJ. Increasing radiation therapy dose is associated with improved survival in patients undergoing stereotactic body radiation therapy for stage I non-small-cell lung cancer. Int J Radiat Oncol Biol Phys (2015) 91(2):344–50. 10.1016/j.ijrobp.2014.10.002 25636759

[51] Videtic GM Paulus R Singh AK Chang JY Parker W Olivier KR Long-term follow-up on NRG oncology RTOG 0915 (NCCTG N0927): a randomized phase 2 study comparing 2 stereotactic body radiation therapy schedules for medically inoperable patients with stage I peripheral non-small cell lung cancer. Int J Radiat Oncol Biol Phys (2019) 103(5):1077–84. 10.1016/j.ijrobp.2018.11.051 30513377 PMC6454873

[52] Singh AK Gomez-Suescun JA Stephans KL Bogart JA Hermann GM Tian L One versus three fractions of stereotactic body radiation therapy for peripheral stage I to II non-small cell lung cancer: a randomized, multi-institution, phase 2 trial. Int J Radiat Oncol Biol Phys (2019) 105(4):752–9. 10.1016/j.ijrobp.2019.08.019 31445956 PMC7043929

[53] Tjong MC Louie AV Singh AK Videtic G Stephans K Plumridge N Single-fraction stereotactic ablative body radiotherapy to the lung – the knockout punch. Clin Oncol (2022) 34(5):e183–94. 10.1016/j.clon.2022.02.004 35221140

[54] Guckenberger M Belka C Bezjak A Bradley J Daly ME DeRuysscher D Practice recommendations for lung cancer radiotherapy during the COVID-19 pandemic: an ESTRO-ASTRO consensus statement. Radiother Oncol (2020) 146:223–9. 10.1016/j.radonc.2020.04.001 32342863 PMC7252074

[55] Park C Papiez L Zhang S Story M Timmerman RD. Universal survival curve and single fraction equivalent dose: useful tools in understanding potency of ablative radiotherapy. Int J Radiat Oncol Biol Phys (2008) 70(3):847–52. 10.1016/j.ijrobp.2007.10.059 18262098

[56] Guckenberger M Klement RJ Allgäuer M Appold S Dieckmann K Ernst I Applicability of the linear-quadratic formalism for modeling local tumor control probability in high dose per fraction stereotactic body radiotherapy for early stage non-small cell lung cancer. Radiother Oncol (2013) 109(1):13–20. 10.1016/j.radonc.2013.09.005 24183066

[57] Wang B Dong Y Yu X Li F Wang J Chen H A review on the applications of Traditional Chinese medicine polysaccharides in drug delivery systems. Front Oncol (2022) 17:12. 10.1186/s13020-021-00567-3 PMC8760834 35033122

[58] Verma V Shostrom VK Kumar SS Zhen W Hallemeier CL Braunstein SE Multi-institutional experience of stereotactic body radiotherapy for large (≥5 centimeters) non–small cell lung tumors. Cancer (2017) 123(4):688–96. 10.1002/cncr.30375 27741355 PMC10905610

[59] Benedict SH Yenice KM Followill D Galvin JM Hinson W Kavanagh B Stereotactic body radiation therapy: the report of AAPM Task Group 101. Med Phys (2010) 37:4078–101. 10.1118/1.3438081 20879569

[60] Keall PJ Mageras GS Balter JM Emery RS Forster KM Jiang SB The management of respiratory motion in radiation oncology report of AAPM Task Group 76. Med Phys (2006) 33:3874–900. 10.1118/1.2349696 17089851

[61] Caillet V Booth JT Keall P. IGRT and motion management during lung SBRT delivery. Physica Med (2017) 44:113–22. 10.1016/j.ejmp.2017.06.006 28647449

[62] Jin JY Ajlouni M Chen Q Kong FM Ryu S Movsas B. Quantification of incidental dose to potential clinical target volume (CTV) under different stereotactic body radiation therapy (SBRT) techniques for non-small cell lung cancer - tumor motion and using internal target volume (ITV) could improve dose distribution in CTV. Radiother Oncol (2007) 85(2):267–76. 10.1016/j.radonc.2007.09.004 17905457

[63] Marks LB Yorke ED Jackson A Ten Haken RK Constine LS Eisbruch A Use of normal tissue complication probability models in the clinic. Int J Radiat Oncol Biol Phys (2010) 76(3):S10–S19. 10.1016/j.ijrobp.2009.07.1754 20171502 PMC4041542

[64] Diez P Hanna GG Aitken KL van As N Carver A Colaco RJ UK 2022 consensus on normal tissue dose-volume constraints for oligometastatic, primary lung and hepatocellular carcinoma stereotactic ablative radiotherapy. Clin Oncol (2022) 34(5):288–300. 10.1016/j.clon.2022.02.010 35272913

[65] Grimm J Marks LB Jackson A Kavanagh BD Xue J Yorke E. High dose per fraction, hypofractionated treatment effects in the clinic (HyTEC): an overview. Int J Radiat Oncol Biol Phys (2021) 110(1):1–10. 10.1016/j.ijrobp.2020.10.039 33864823 PMC9447432

[66] Verbakel WFAR Senan S Cuijpers JP Slotman BJ Lagerwaard FJ. Rapid delivery of stereotactic radiotherapy for peripheral lung tumors using volumetric intensity-modulated arcs. Radiother Oncol (2009) 93(1):122–4. 10.1016/j.radonc.2009.05.020 19552979

[67] Navarria P Ascolese AM Mancosu P Alongi F Clerici E Tozzi A Volumetric modulated arc therapy with flattening filter free (FFF) beams for stereotactic body radiation therapy (SBRT) in patients with medically inoperable early stage non small cell lung cancer (NSCLC). Radiother Oncol (2013) 107(3):414–8. 10.1016/j.radonc.2013.04.016 23725859

[68] De Ruysscher D Belderbos J Reymen B Van Elmpt W Van Baardwijk A Wanders R State of the art radiation therapy for lung cancer 2012: a glimpse of the future. Clin Lung Cancer (2013) 14:89–95. 10.1016/j.cllc.2012.06.006 22853981

[69] Devaraj A Cook GJR Hansell DM. PET/CT in non-small cell lung cancer staging-promises and problems. Clin Radiol (2007) 62:97–108. 10.1016/j.crad.2006.09.015 17207691

[70] Cheebsumon P Boellaard R de Ruysscher D van Elmpt W van Baardwijk A Yaqub M Assessment of tumour size in PET/CT lung cancer studies: PET- and CT-based methods compared to pathology. EJNMMI Res (2012) 2(1):56–9. 10.1186/2191-219X-2-56 23034289 PMC3502476

[71] Geiger GA Kim MB Xanthopoulos EP Pryma DA Grover S Plastaras JP Stage migration in planning PET/CT scans in patients due to receive radiotherapy for non-small-cell lung cancer. Clin Lung Cancer (2014) 15(1):79–85. 10.1016/j.cllc.2013.08.004 24238934

[72] Loganadane G Martinetti F Mercier O Krhili S Riet FG Mbagui R Stereotactic ablative radiotherapy for early stage non-small cell lung cancer: a critical literature review of predictive factors of relapse. Cancer Treat Rev (2016) 50:240–6. 10.1016/j.ctrv.2016.10.002 27768919

[73] Chang JY Liu H Balter P Komaki R Liao Z Welsh J Clinical outcome and predictors of survival and pneumonitis after stereotactic ablative radiotherapy for stage I non-small cell lung cancer. Radiat Oncol (2012) 7(1):152. 10.1186/1748-717X-7-152 22963661 PMC3444889

[74] Vansteenkiste J Crinò L Dooms C Douillard JY Faivre-Finn C Lim E 2nd ESMO consensus conference on lung cancer: early-stage non-small-cell lung cancer consensus on diagnosis, treatment and follow-up. Ann Oncol (2014) 25(8):1462–74. 10.1093/annonc/mdu089 24562446

[75] Videtic GMM Donington J Giuliani M Heinzerling J Karas TZ Kelsey CR Stereotactic body radiation therapy for early-stage non-small cell lung cancer: executive Summary of an ASTRO Evidence-Based Guideline. Pract Radiat Oncol (2017) 7(5):295–301. 10.1016/j.prro.2017.04.014 28596092

[76] Zeng J Harris TJ Lim M Drake CG Tran PT. Immune modulation and stereotactic radiation: improving local and abscopal responses. Biomed Res Int (2013) 2013:658126. 10.1155/2013/658126 24324970 PMC3845488

[77] Chen Y Gao M Huang Z Yu J Meng X. SBRT combined with PD-1/PD-L1 inhibitors in NSCLC treatment: a focus on the mechanisms, advances, and future challenges. J Hematol Oncol (2020) 13:105. 10.1186/s13045-020-00940-z 32723363 PMC7390199

[78] Shaverdian N Lisberg AE Bornazyan K Veruttipong D Goldman JW Formenti SC Previous radiotherapy and the clinical activity and toxicity of pembrolizumab in the treatment of non-small-cell lung cancer: a secondary analysis of the KEYNOTE-001 phase 1 trial. Lancet Oncol (2017) 18(7):895–903. 10.1016/S1470-2045(17)30380-7 28551359 PMC5538772

[79] Wang YY Wang YS Liu T Yang K Yang GQ Liu HC Efficacy study of CyberKnife stereotactic radiosurgery combined with CIK cell immunotherapy for advanced refractory lung cancer. Exp Ther Med (2013) 5(2):453–6. 10.3892/etm.2012.818 23403795 PMC3570163

[80] Seung SK Curti BD Crittenden M Walker E Coffey T Siebert JC Phase 1 study of stereotactic body radiotherapy and interleukin-2--tumor and immunological responses. Sci Transl Med (2012) 4(137):137ra74. 10.1126/scitranslmed.3003649 22674552

[81] Monjazeb AM Daly ME Luxardi G Maverakis E Merleev AA Marusina AI Atezolizumab plus stereotactic ablative radiotherapy for medically inoperable patients with early-stage non-small cell lung cancer: a multi-institutional phase I trial. Nat Commun (2023) 14(1):5332. 10.1038/s41467-023-40813-w 37658083 PMC10474145

[82] Huang K Dahele M Senan S Guckenberger M Rodrigues GB Ward A Radiographic changes after lung stereotactic ablative radiotherapy (SABR) - can we distinguish recurrence from fibrosis? A systematic review of the literature. Radiother Oncol (2012) 102:335–42. 10.1016/j.radonc.2011.12.018 22305958

[83] Bucknell NW Kron T Herschtal A Hardcastle N Irving L MacManus M Comparison of changes in pulmonary function after stereotactic body radiation therapy versus conventional 3-dimensional conformal radiation therapy for stage I and IIA non-small cell lung cancer: an analysis of the TROG 09.02 (CHISEL) phase 3 trial. Int J Radiat Oncol Biol Phys (2023) 117(2):378–86. 10.1016/j.ijrobp.2023.04.009 37087060

[84] Yu V Kishan AU Cao M Low D Lee P Ruan D. Dose impact in radiographic lung injury following lung SBRT: statistical analysis and geometric interpretation. Med Phys (2014) 41(3):031701. 10.1118/1.4863483 24593705

[85] Stanic S Paulus R Timmerman RD Michalski JM Barriger RB Bezjak A No clinically significant changes in pulmonary function following stereotactic body radiation therapy for early-stage peripheral non-small cell lung cancer: an analysis of RTOG 0236. Int J Radiat Oncol Biol Phys (2014) 88(5):1092–9. 10.1016/j.ijrobp.2013.12.050 24661663 PMC4058437

[86] Finazzi T Palacios MA Spoelstra FOB Haasbeek CJA Bruynzeel AME Slotman BJ Role of on-table plan adaptation in MR-guided ablative radiation therapy for central lung tumors. Int J Radiat Oncol Biol Phys (2019) 104(4):933–41. 10.1016/j.ijrobp.2019.03.035 30928360

[87] Finazzi T Palacios MA Haasbeek CJA Admiraal MA Spoelstra FOB Bruynzeel AME Stereotactic MR-guided adaptive radiation therapy for peripheral lung tumors. Radiother Oncol (2020) 144:46–52. 10.1016/j.radonc.2019.10.013 31710943

[88] Henke LE Olsen JR Contreras JA Curcuru A DeWees TA Green OL Stereotactic MR-guided online adaptive radiation therapy (SMART) for ultracentral thorax malignancies: results of a phase 1 trial. Adv Radiat Oncol (2019) 4(1):201–9. 10.1016/j.adro.2018.10.003 30706029 PMC6349650

